# ‘Nowhere and no one is safe’: spatial analysis of damage to critical civilian infrastructure in the Gaza Strip during the first phase of the Israeli military campaign, 7 October to 22 November 2023

**DOI:** 10.1186/s13031-024-00580-x

**Published:** 2024-04-02

**Authors:** Yara Asi, David Mills, P. Gregg Greenough, Dennis Kunichoff, Saira Khan, Jamon Van Den Hoek, Corey Scher, Saleem Halabi, Sawsan Abdulrahim, Nadine Bahour, A. Kayum Ahmed, Bram Wispelwey, Weeam Hammoudeh

**Affiliations:** 1 FXB Center for Health and Human Rights, Harvard University, Boston, USA; 2 School of Global Health Management and Informatics, University of Central Florida, Orlando, USA; 3 University of California San Diego School of Medicine, La Jolla, USA; 4 Harvard Humanitarian Initiative, Harvard University, Cambridge, USA; 5 Harvard Medical School, Boston, USA; 6 College of Earth, Ocean, and Atmospheric Sciences (CEOAS), Oregon State University, Corvallis, USA; 7 The Graduate Center, City University of New York, New York, USA; 8 Faculty of Health Sciences, American University of Beirut, Beirut, Lebanon; 9 Columbia University Mailman School of Public Health, New York, USA; 10 Institute of Community and Public Health, Birzeit University, Birzeit, Palestine

**Keywords:** Israel, Gaza Strip, Israel-Hamas war, Health, Education, Water, Civilian infrastructure, Humanitarian, International Humanitarian Law, Spatial analysis

## Abstract

**Background:**

Since the Hamas attacks in Israel on 7 October 2023, the Israeli military has launched an assault in the Gaza Strip, which included over 12,000 targets struck and over 25,000 tons of incendiary munitions used by 2 November 2023. The objectives of this study include: (1) the descriptive and inferential spatial analysis of damage to critical civilian infrastructure (health, education, and water facilities) across the Gaza Strip during the first phase of the military campaign, defined as 7 October to 22 November 2023 and (2) the analysis of damage clustering around critical civilian infrastructure to explore broader questions about Israel’s adherence to International Humanitarian Law (IHL).

**Methods:**

We applied multi-temporal coherent change detection on Copernicus Sentinel 1-A Synthetic Aperture Radar (SAR) imagery to detect signals indicative of damage to the built environment through 22 November 2023. Specific locations of health, education, and water facilities were delineated using open-source building footprint and cross-checked with geocoded data from OCHA, OpenStreetMap, and Humanitarian OpenStreetMap Team. We then assessed the retrieval of damage at and with close proximity to sites of health, education, and water infrastructure in addition to designated evacuation corridors and civilian protection zones. The Global Moran’s I autocorrelation inference statistic was used to determine whether health, education, and water facility infrastructure damage was spatially random or clustered.

**Results:**

During the period under investigation, in the entire Gaza Strip, 60.8% (*n* = 59) of health, 68.2% (*n* = 324) of education, and 42.1% (*n* = 64) of water facilities sustained infrastructure damage. Furthermore, 35.1% (*n* = 34) of health, 40.2% (*n* = 191) of education, and 36.8% (*n* = 56) of water facilities were functionally destroyed. Applying the Global Moran’s I spatial inference statistic to facilities demonstrated a high degree of damage clustering for all three types of critical civilian infrastructure, with Z-scores indicating < 1% likelihood of cluster damage occurring by random chance.

**Conclusion:**

Spatial statistical analysis suggests widespread damage to critical civilian infrastructure that should have been provided protection under IHL. These findings raise serious allegations about the violation of IHL, especially in light of Israeli officials’ statements explicitly inciting violence and displacement and multiple widely reported acts of collective punishment.

**Supplementary Information:**

The online version contains supplementary material available at 10.1186/s13031-024-00580-x.

## Background

The Gaza Strip is a small coastal enclave with the Mediterranean Sea to the west, Egypt to the south, and Israel to the north and east, although it has no legally defined borders. It is part of the occupied Palestinian territory (oPt), along with the West Bank and East Jerusalem, to which it is not geographically connected. Home to approximately 2.2 million people as of 2022 [1], the Gaza Strip is commonly recognized as one of the most densely populated places in the world (approximately 15,000 persons/sq mile) [2].

On 7 October 2023, Hamas militants launched an attack inside Israel killing approximately 1200 people and taking 240 hostages [3]. Almost immediately, Israel launched a wide-scale military campaign on the Gaza Strip and instituted a complete siege on all its land borders. At the time of writing, in late February 2024, Israel’s military campaign has killed more than 29,000 people in the Gaza Strip, around 70% of whom are estimated to be women and children [4]. The first 46 days included an unprecedented civilian death toll, eclipsing the total number of Ukrainian civilian deaths in the first 21 months of the Russia-Ukraine War that began in 2022 [5].

The Gaza Strip has experienced four other massive Israeli military assaults over the past 15 years. In each instance, widespread damage to homes, businesses, utility infrastructure, and educational and health facilities has been documented, leading to warnings of potential violations of the laws of war by Israel [6–9]. In the first month of the current military campaign, over 25,000 tons of incendiary munitions have been fired into the Gaza Strip [10], including two 900-kilogram bombs on the densely populated Jabalia refugee camp on 31 October 2023 [11]. The Israeli military struck over 12,000 targets with extensive human consequences. By the end of 2023, over 1.9 million Palestinians in Gaza (85% of the total population) had been internally displaced due to bombing and evacuation orders [12].

Since the outset of the 2023 military campaign, there has been regular reporting and subsequent international condemnation of the widespread damage and destruction of infrastructure throughout the Gaza Strip [13, 14]. Such incidents are especially concerning considering statements by several Israeli officials that dehumanize Palestinians, incite violence against them, or call for their displacement [15]. The high death toll and level of damage in the Gaza Strip have raised serious allegations of the violation of International Humanitarian Law (IHL) by the Israeli military; specifically, whether Israel’s bombings “distinguish between the civilian population and combatants, and between civilian objects and military objectives” and if the “incidental harm on civilians is proportional to the concrete and direct military advantage anticipated.” [16] These questions become especially relevant when taken into account other Israeli actions during this military campaign, including instituting a complete siege of food, fuel, water, and medicine [17]; conducting mass arrests of men and boys [18]; vocal campaigns by Israeli politicians for resettlement of residents of Gaza to other countries [19]; the massive forced displacement from the north to the south of Gaza [20]; and other actions that have been called war crimes by Human Rights Watch [21], Amnesty International [22], and other human rights groups [23].

IHL offers specific protections to civilian infrastructure, such as schools and hospitals. Parties to an armed conflict must at all times distinguish between civilians and civilian objects on the one hand, and soldiers and military objectives on the other. Direct or indiscriminate attacks on civilians and civilian objects are prohibited. A hospital or school may become a legitimate military target only if it is both being used for specific military operations of the enemy and also if its destruction offers a defined military advantage. According to the International Committee of the Red Cross, “If there is any doubt, they cannot be attacked.” [24].

When attacking a military objective, parties are obligated to take all necessary precautions to avoid, or at the very least, minimize, death and injury to civilians and damage to civilian objects. Such precautions include doing everything possible to verify that a target is a military objective; selecting methods of attack that minimize civilian harm; assessing whether an attack would be disproportionate; giving effective advance warning; and canceling an attack if it becomes apparent that such an attack would be unlawful.

This study provides a descriptive and inferential spatial assessment of damage to critical civilian infrastructure across the Gaza Strip during the first phase of the military campaign, from 7 October to 22 November 2023, two days before the temporary ceasefire went into effect. The primary objective of the study is to describe patterns of damaged infrastructure and the proximity to critical civilian infrastructure, defined in this study as hospitals and health centers (health facilities), universities and schools (education facilities), water storage and access points (water facilities), and the designated evacuation corridor. These facilities were chosen because of their clear status as critical civilian infrastructure that is protected under IHL, as infrastructure required for the ability to sustain life in parts of the Gaza Strip, and the availability of geocoded data for these humanitarian sectors. The secondary objective of the study is to utilize spatial statistics to determine damage clustering in order to explore broader questions about whether the war has been waged in a way that adheres to IHL by offering protections to these civilian infrastructures.

## Methods

**Fig. 1:**
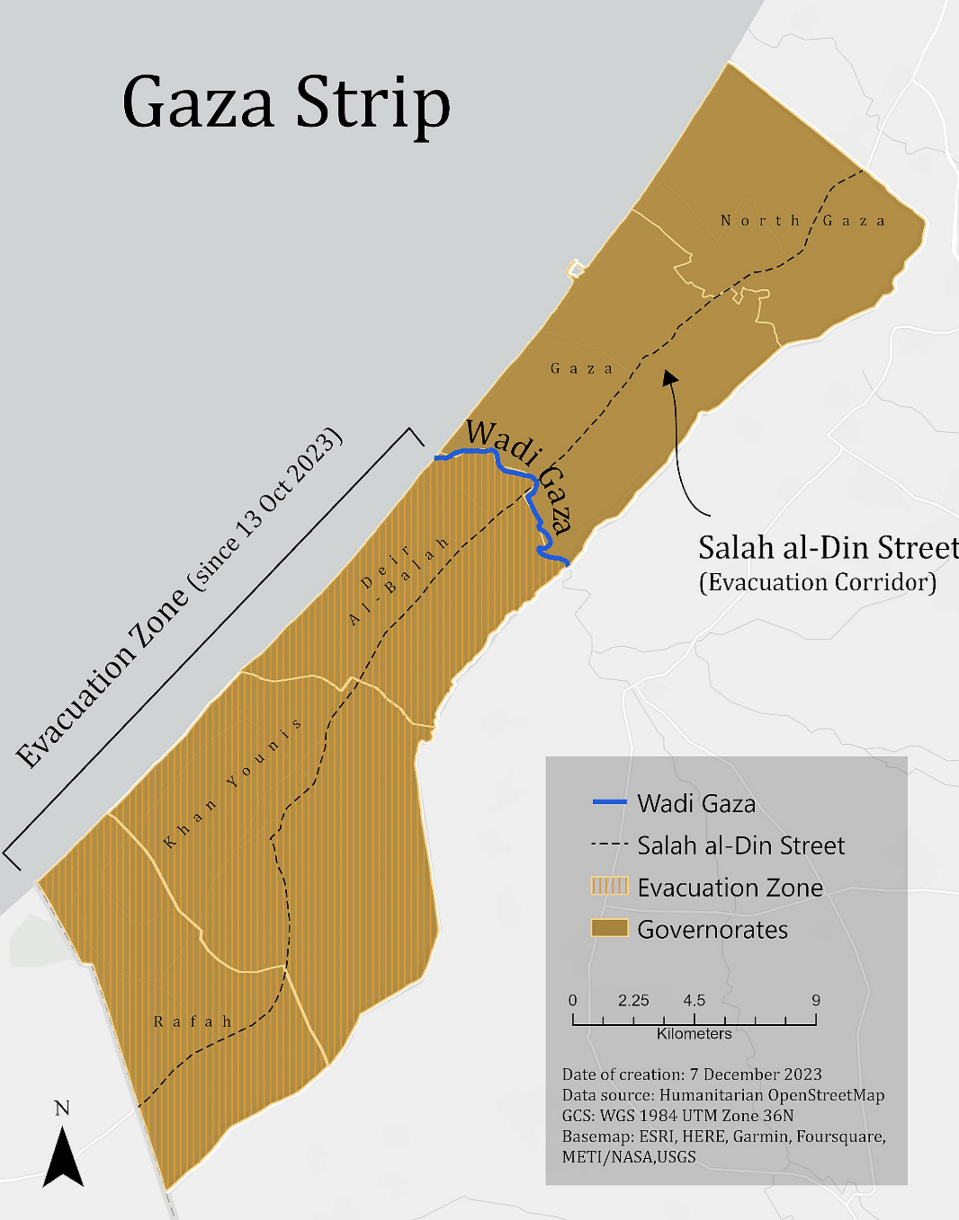
Map of the Gaza Strip outlining five governorates, the Wadi Gaza evacuation zone line, and the Salah al-Din Street evacuation corridor

This paper characterizes the damage of critical civilian infrastructure across all five governorates of the Gaza Strip (Fig. 1) and whether infrastructure damage occurs in a spatially random pattern. The analyzed civilian infrastructure includes facilities protected under IHL and are defined specifically in this study as health, education, and water facilities. The analysis also explores the relationship of infrastructure damage to areas designated as a ‘protected’ evacuation zone and evacuation corridor by the Israeli military evacuation order given on 13 October 2023. This order, which forced the displacement of the two northern Gaza Strip governorate populations south via the Salah al-Din Street southwest vectored corridor, past the Wadi Gaza demarcation line to the southern three governorates (Fig. 1), was in effect until 1 December 2023, encompassing the full period of our study.

This study incorporates satellite radar and other open-source datasets to spatially analyze and characterize the damage from the impact of the first phase of the Israeli military campaign on critical civilian infrastructure protected under IHL.

### Satellite radar-based damage analysis

A map of structural damage through 22 November 2023 was made with open-access 10 m resolution European Space Agency Copernicus Sentinel-1 A Synthetic Aperture Radar (SAR) data using a multi-temporal coherent change detection approach [25]. Coherent change detection has wide uptake in satellite radar-based approaches to map earthquake damage (e.g. NASA ARIA emergency response data products), and rests on the measurement of changes in coherence, which is the similarity between phase components of interferometric radar waves collected over different points in time [26]. Stable coherence over time in a built-up environment like the Gaza Strip suggests the persistent presence of a feature, such as a building [27], while a large and persistent decrease in coherence from one image date to the next suggests structural damage or destruction [28, 29]. Native resolution Sentinel-1 data acquired in the interferometric-wide swath mode have a spatial resolution of 5 m by 20 m in range and azimuth (radar geometries), which, when projected onto a geospatial grid in ground range, results in a 10 m pixel spacing product. To conduct time series analysis of coherence data from Sentinel-1, native resolution data requires interferometric processing, which results in a 40 m pixel spacing grid for the coherence data used in this study. Interferometric processing of Sentinel-1 data was conducted using the Alaska Satellite Facility’s HyP3 cloud-based processing infrastructure [30].

To map locations of likely damage, a one-year baseline period (2022 to 2023) was established to identify regions with high magnitude and low variability of coherence over time. Regions with sufficient stability (high coherence, low variability) during the baseline period were identified, and changes in coherence were tracked after 7 October 2023. For every identified stable pixel in each Sentinel-1 A radar image of the Gaza Strip acquired thereafter, the change in coherence was measured relative to the baseline coherence and locations of statistically significant decreases in coherence were recorded as likely damage. Using this approach, a map of cumulative likely damage through 22 November 2023 was produced. The area of damage was recorded for all building footprints documented in the Microsoft building footprints or OpenStreetMap (OSM) datasets (Table 1). The resulting structure-level cumulative likely damage estimates form the basis for our damage assessments described below. (Since ground truth validation data were not available to confirm the presence of structural damage indicated by satellite radar analysis and thus precludes quantification of damage accuracy, the study’s authors found a moderately high agreement with a damage map produced by UNOSAT [31]. Using a total likely damage map across the Gaza Strip based on radar data acquired through 5 November 2023, we were able to detect 68% of building damage locations reported by UNOSAT based on analysis of very-high resolution 30 cm WorldView-3 commercial satellite imagery collected on 7 November 2023. (This level of agreement is especially notable given that our analysis is based on 10-meter resolution data).

### Spatial analysis of damage of health, education, and water facilities infrastructure

Pre-7 October georeferenced datasets on health facilities, education facilities, and water facilities were imported, modified, and analyzed in ArcGIS Pro 3.2 (ESRI, Redlands, CA) and R. Point data on health facilities, which included hospitals and all types of specialty clinics, were from the UN Office for the Coordination of Humanitarian Affairs (OCHA); a georeferenced dataset capturing education facilities, including kindergartens, schools, colleges, and universities, was generated using point data from OSM and Humanitarian OpenStreetMap (HOTOSM) (Table 1).

Georeferenced water facility point data was also from HOTOSM. The research team generated polygons from the imagery pixels to capture the entire footprint of the educational facilities and health facilities based on the imported point data. Confirmed with very high-resolution satellite imagery and OSM base map data, the resulting polygon dataset was reviewed for completeness and redundancy against available data from HOTOSM, and a 5-meter buffer was generated across each health and education facility polygon and water point to improve precision and minimize georeferencing errors.

**Table 1:** Data sources

	Data Source	Selection criteria
Footprints	https://github.com/microsoft/GlobalMLBuildingFootprints https://openstreetmap.org	NA
Health	https://data.humdata.org/dataset/state-of-palestine-health-0	Clinics and hospitals
Education	https://data.humdata.org/dataset/state-of-palestine-schools https://data.humdata.org/dataset/hotosm_pse_education_facilities	Kindergartens, schools, colleges, and universities
Water	https://data.humdata.org/dataset/hotosm_pse_points_of_interest	Water distribution or storage sites labeled as “water well”, “water tap”, “drinking water”, and “water point”

To capture the extent of damage to health, education, and water facilities, the number of each type of facility polygon with a non-zero area of cumulative damage was recorded. Next, 25-meter and 50-meter buffers were generated around these facility polygons as well as the evacuation corridor to measure damage in immediate proximity to health, education, and water facility polygons. The widths of these buffers were defined by the lethality and blast radii of the predominant incendiary weapons used by the Israeli military in the Gaza Strip: the precision-guided MK-80 series, whose sizes range between 120 and 1000 kg [32] and 155 mm surface artillery shells. The 500 kg MK-82, with 89 kg of high explosives, has a high velocity blast of 32 m diameter, with peak overpressures extending to 31 m radius (62 m diameter), collapsing most buildings, severely damaging heavy concrete structures, injuring nearly everyone within that diameter, and killing most [33]. The 155 mm artillery shells have a lethality radius of 50 m and injury radius of 150 m [34]. We considered the 25 and 50 m buffers as conservative estimates given that the fragmentation of these weapons upon detonation can extend much further than nominally reported, and that the US Office of the Director of National Intelligence (ODNI) Assessment found 40–45% of the 29,000 air-to-ground munitions Israel has dropped in the Gaza Strip have been unguided and with less precise and less discriminate targeting compared to precision-guided weapons [35] which theoretically have more civilian protecting capacity. We measured the area and calculated the percent of damage to each of the health and education facility polygons and the water point 5 m buffer polygons, and used the percent of damage to create an indicator variable identifying facilities with at least 50% of the polygons being damaged. A 50% area threshold was chosen based on military damage assessment guidance that considers a building unusable and functionally destroyed once it has sustained 50% structural damage [36].

The degree of spatial relatedness of structural damage at infrastructure sites was also measured using Global Moran’s I. Global Moran’s I is a statistical measure of spatial autocorrelation, which describes whether a geographic pattern of interest, in this case, structural damage at infrastructure locations, is spatially clustered, randomly distributed, or dispersed [37]. Global Moran’s I is calculated using the equation:

$$I = \frac{N}{W}\sum i\frac{{\sum j{w_{ij}}({x_i} - \bar x)({x_j} - \bar x)}}{{\sum i{{({x_i} - \bar x)}^2}}}$$

where N is the number of observations at locations *i* and *j; x* and $\bar x$ are the attribute values of interest (percent of facility polygon damage) and their means, respectively; *w*_*ij*_ is a matrix of spatial weights (the strengths of the spatial relationships in the data set), and W is the sum of all *w*_*ij*_*.* Assuming a null hypothesis of spatial randomness (zero spatial autocorrelation), the expected value of Global Moran’s I is:

$$E \left(I\right)=\frac{-1}{N-1}$$

Positive values for the Global Moran’s I associated with statistically significant *p-*values, defined as below a 0.05 threshold, suggest clustering patterns of infrastructural damage and a rejection of spatial randomness (negligible values) or dispersion (negative values). We measured Global Moran’s I of damage at 25 m buffered infrastructure polygons, used inverse distance weighting, given by 1/*d* where *d* is the distance to *i*, to determine the influence of neighboring locations, Euclidean distance as the measurement construct, and row standardization to minimize sampling design bias.

## Results

**Fig. 2:**
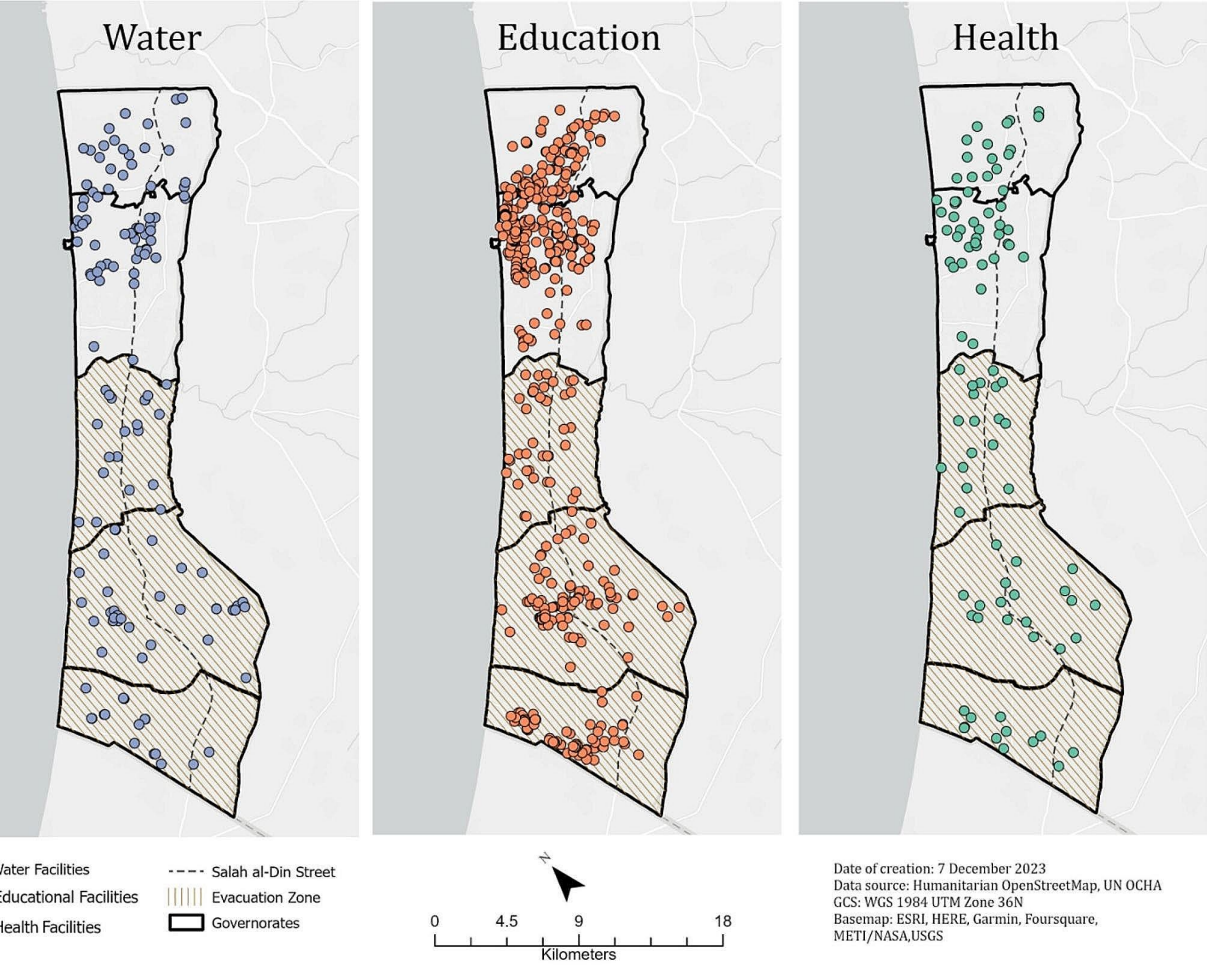
Distribution of health, education, and water facilities infrastructure sites in the Gaza Strip

Figure 2; Table 2 present the number and spatial distribution of facility types stratified by governorate for the analysis, and included 97 health facilities, 475 education facilities, and 152 water facilities. The Gaza governorate, which contains Gaza City, accounted for the largest number of health, education, and water facilities.

**Table 2:** Number of facility types and their distribution across Gaza Strip governorates

Facility Type	Total (N)	North Gaza	Gaza	Deir Al-Balah	Khan Younis	Rafah
Health facilities	97	17 (17.5%)	32 (33%)	18 (18.6%)	18 (18.6%)	12 (12.4%)
Education facilities	475	87 (18.3%)	199 (41.9%)	39 (8.2%)	85 (17.9%)	65 (13.7%)
Water facilities	152	33 (21.7%)	45 (29.6%)	21 (13.8%)	36 (23.7%)	17 (11.2%)

### Infrastructure damage analysis

Our analysis shows that many health, education, and water facilities are directly located or within close proximity to areas of SAR-indicated damage. Damage was detected at over 60% of all health facilities (60.8%, *n* = 59), over two-thirds of education facilities (68.2%, *n* = 324), and over 40% of all sites of water infrastructure (42.1%, *n* = 64) (Table 3). Considering the ‘functional destruction’ threshold where the area of damage is at least half of the area of a facility polygon, we found that 35.1% (*n* = 34) of health facilities, 40.2% (*n* = 191) of education facilities, and 36.8% (*n* = 56) of water facilities were functionally destroyed (Table 3). When assessed for proximity to damage-affected areas, our analysis found evidence of SAR-detected damage within 25 m of 70.1% (*n* = 68) of health facilities, 75.8% (*n* = 360) of education facilities, and 51.3% (*n* = 78) of water facilities in the Gaza Strip; similarly, SAR-detected damage within 50 m was found for 78.4% (*n* = 76) of health facilities, 82.5% (*n* = 392) of education facilities and 58.6% (*n* = 89) of water facilities.

**Table 3:** Percent and counts of damaged facilities in the Gaza Strip by facility type, stratified by whether more or less than half of each facility’s boundary intersected with SAR-detected damage

		Total Facilities	Damage		
Buffer level (meters)	Facility Type	*N*	Any damage	< 50%	>=50%
0	Health facilities	97	59 (60.8%)	25 (25.8%)	34 (35.1%)
25			68 (70.1%)	41 (42.3%)	27 (27.8%)
50			76 (78.4%)	49 (50.5%)	27 (27.8%)
0	Education facilities	475	324 (68.2%)	133 (28%)	191 (40.2%)
25			360 (75.8%)	199 (41.9%)	161 (33.9%)
50			392 (82.5%)	243 (51.2%)	149 (31.4%)
0	Water facilities	152	64 (42.1%)	8 (5.3%)	56 (36.8%)
25			78 (51.3%)	17 (11.2%)	61 (40.1%)
50			89 (58.6%)	31 (20.4%)	58 (38.2%)

**Fig. 3:**
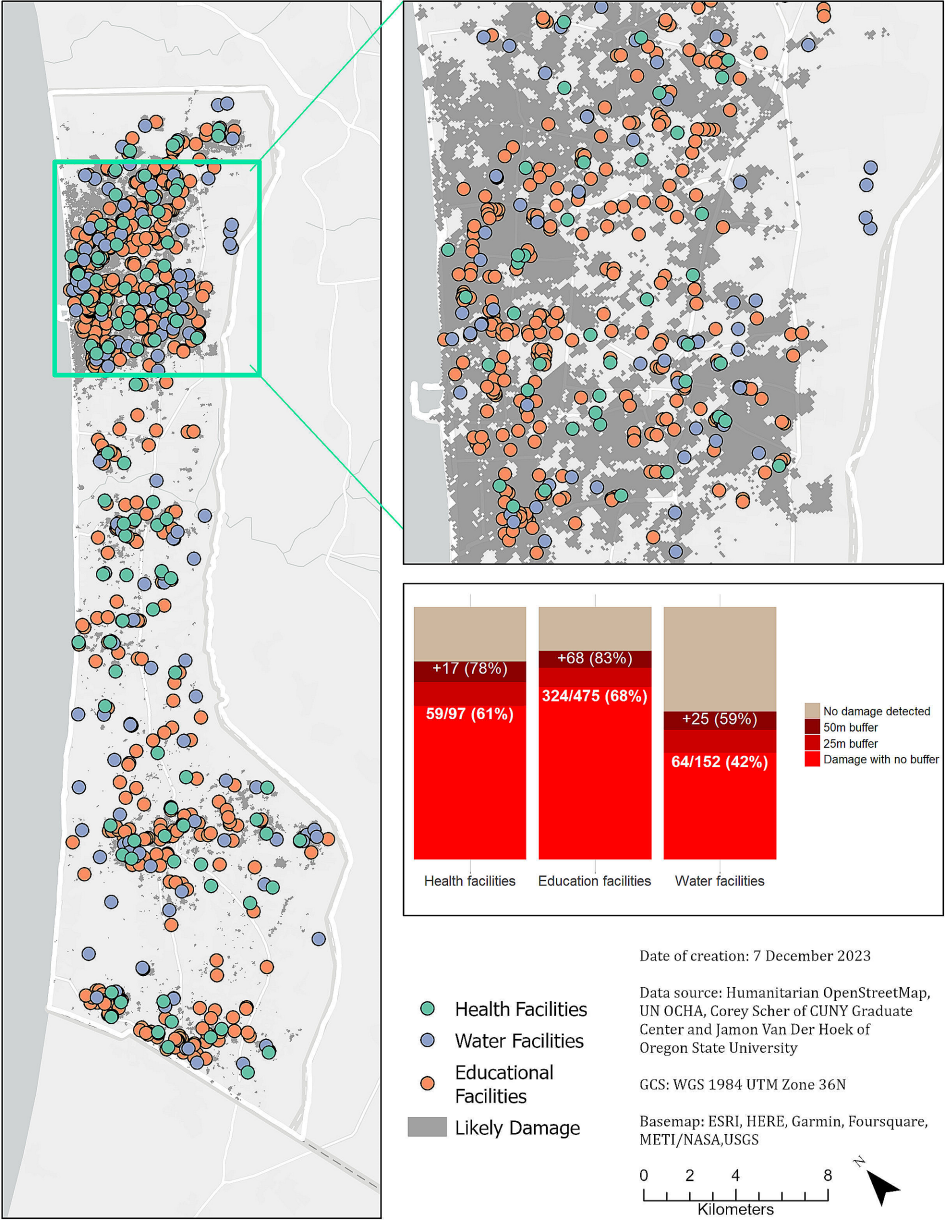
Map of health, education, and water facilities overlaying cumulative damage, with an inset map in Gaza City, and the corresponding areas of damage for each facility type within each radial buffer across the Gaza Strip

Figure 3 illustrates the cumulative burden of infrastructure damage across the Gaza Strip through 22 November 2023, and the spatial relationship of that infrastructure damage to health, education, and water facilities. Figure 3 also outlines the designated evacuation corridor and evacuation zone of the southern governorates. The majority of the damage in this first phase of the military campaign took place in the northern two governorates which contains Gaza City, the most populated city in the Gaza Strip.

**Fig. 4:**
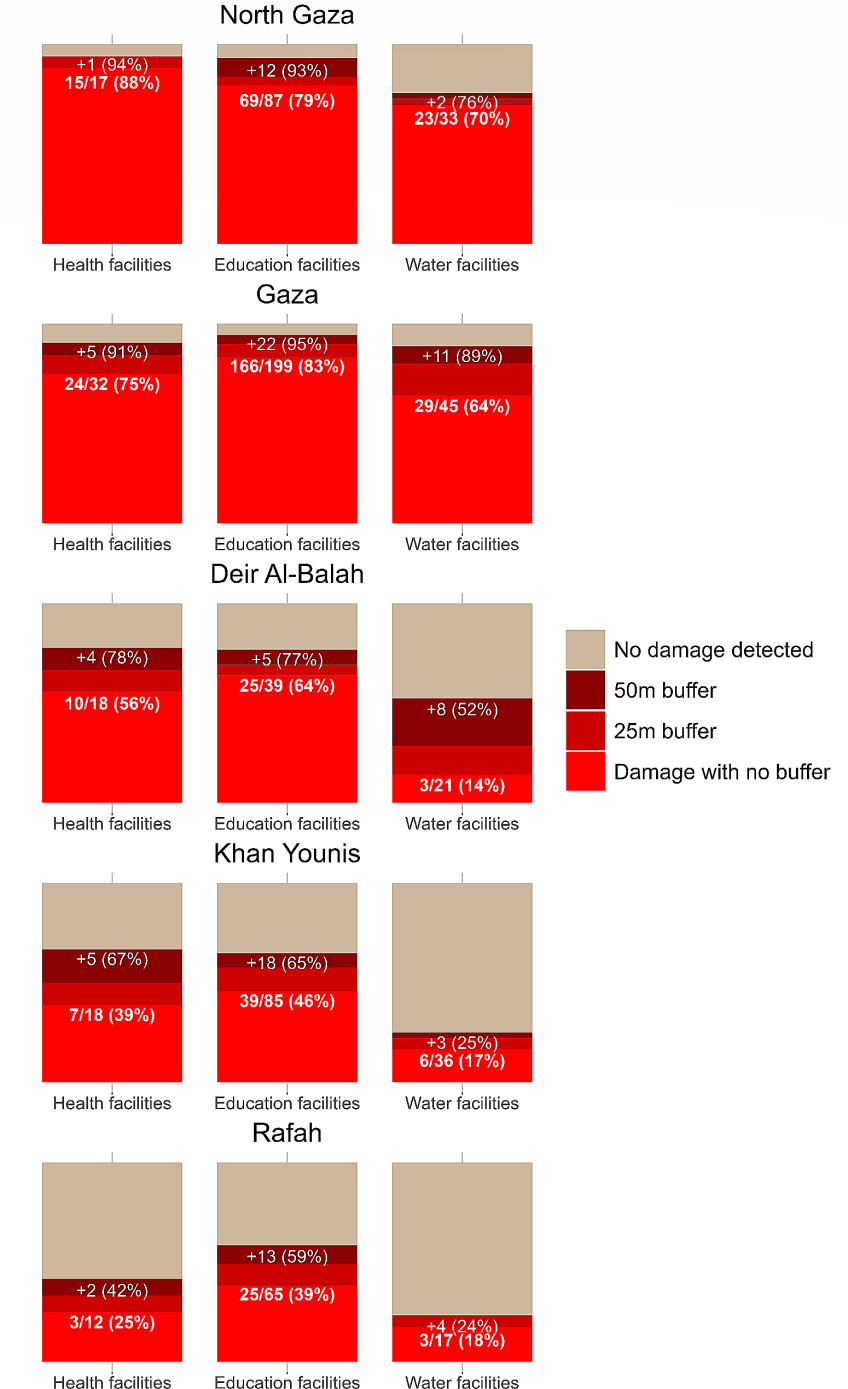
Number of damaged facilities and percent damage to health, education, and water facilities by governorate and buffered area

The degree of damage by governorate and facility type is reported in Fig. 4. In North Gaza, 88.2% (*n* = 15) of health facilities, 79.3% (*n* = 69) of education facilities, and 70% (*n* = 23) of water facilities sustained direct damage. In the Gaza City governorate, 75% (*n* = 24) of health facilities, 83.4% (*n* = 166) of education facilities, and 64.4% (*n* = 29) of water facilities sustained direct damage. When assessing facilities that were functionally destroyed, 57.1.% (*n* = 28) of health facilities, 58.4% (*n* = 167) of education facilities, and 57.7% (*n* = 45) of water facilities in North Gaza and Gaza City governorates met the 50% or more damage threshold. In the three southern governorates designated the evacuation zone below the Wadi Gaza, 41.7% (*n* = 20) of the health facilities, 47.1% (*n* = 89) of the education facilities, and 16.2% (*n* = 12 of the water facilities had direct damage, and 12.5% (*n* = 6) of health facilities, 12.7% (*n* = 24) of education facilities, and 14.9% (*n* = 11) of water facilities were functionally destroyed. Details for the counts and percentages of damaged facilities at all three buffer levels [0 m, 25 m, and 50 m] and counts stratified by the equal to or greater than 50% damage threshold for each governorate can be found in Supplemental Table 1.

The line representation of the Salah Al-Din Street evacuation corridor that we generated for this analysis was 46.8 km in total length and was used to assess proximity to damage at the three buffer levels (Table 4). 2.1% of the evacuation corridor intersected directly with damage. This proportion of the evacuation corridor represents 1 km of its total length. The evacuation corridor with the 50 m buffer intersected with damage at 11.6% of the road, 5 km of its total length.

**Table 4:** Proximity of damage along the evacuation corridor of Salah al-Din Street, 7 October to 22 November 2023

Salah al-Din Street	
Buffer level (meters)	Percent damage
0	2.1%
25	8.4%
50	11.6%

### Spatial statistical analysis

Applying Global Moran’s I - a spatial inference statistic to discern spatial heterogeneity - to the dataset of health, education, and water facilities across the Gaza Strip, we found a high degree of damage clustering for all three types of critical civilian infrastructure, above and below the 50% damage threshold (Table 5). For all the facilities studied, Z-scores of these magnitudes indicate a < 1% likelihood that this amount of clustering would occur by random chance, regardless of the degree of damage. This suggests that damage to these structures is highly autocorrelated.

**Table 5:** Spatial autocorrelation of infrastructure damage to facilities in the Gaza Strip by facility type

Facility Type	Global Moran’s I	*p*-value		Z-score
Health facilities				
Any damage >=50% damage	0.380895 0.348284	< 0.000001* < 0.000001*		5.848462* 5.362817*
Education facilities				
Any damage >=50% damage	0.431564 0.342495	< 0.000001* < 0.000001*		19.860574* 15.776496*
Water facilities				
Any damage >=50% damage	0.544802 0.519995	< 0.000001* < 0.000001*		9.837989* 9.385527*

* statistically significant

## Discussion

The findings from this study add to the evidence indicating that the level and scope of damage to critical civilian infrastructure in the Gaza Strip from the first phase of the Israeli military campaign between 7 October and 22 November 2023 has few, if any, precedents in recent conflicts. Within just 46 days, much of the critical civilian infrastructure in the Gaza Strip was damaged or destroyed. Direct damage of facilities across all Gaza Strip governorates included over half of health and education facilities and over a third of the water facilities. In the North and Gaza governorates–the most populated governorates in the Gaza Strip–over half of each facility type was destroyed. Over a third of all health, education, and water facilities across all governorates had equal to or greater than 50% damage, by definition deeming them completely destroyed [36].

Spatial analysis of this infrastructure damage (Table 5) demonstrates a lack of randomness and statistically significant clustering of damage around critical civilian infrastructure. Whereas negative Z-scores would suggest avoidance of such infrastructure, positive Z-scores of these magnitudes indicate a < 1% likelihood that this amount of clustering would occur by random chance, regardless of the degree of damage. The clustering of damage detected at and adjacent to critical civilian infrastructure described by this data demonstrates that not only are these civilian sites not being afforded legal protections as mandated under IHL, but they are being consistently damaged and destroyed, supporting claims that “nowhere and no one is safe” in the Gaza Strip [38]. It is important to note the time-limited period of this analysis. Bombing continued after the temporary ceasefire ended at the end of November, and the ramifications of these additional attacks were not included in this analysis, as this study focused on the period before the temporary ceasefire.

This level of damage raises questions about whether these areas of the Gaza Strip will be able to sustain civilian life once the Israeli military campaign concludes, even for those few whose homes may be left standing. The critical civilian infrastructure necessary for life has been destroyed in many parts of the northern Gaza Strip and will require significant investments in time and funding to be able to sustain communities again. Along with the high civilian death toll [39], such damage has led to the rapid deterioration of the living conditions for survivors of bombings, leaving them unable to access basic services vital for the realization of the right to the highest attainable standard of physical and mental health, well-being, or even basic survival [40–42]. At least 1.3 million internally displaced people were sheltering in 155 United Nations Relief and Works Agency sites by the end of the first stage of the military campaign, frequently within health and education facilities [43, 44], meaning that damage to these buildings represented direct threats to sheltering civilians. The inability of international actors to protect these facilities, despite the overwhelming destruction, demonstrates the fundamental limitations of IHL that should be seen as “intolerable, as states and armed groups can use the fog of war and flexibilities in their targeting assessments to justify virtually any attack.” [45].

While the data from this study cannot determine intentionality, the strength of clustering suggests the possibility of direct attacks on critical civilian infrastructure as part of a larger program of collective punishment, in line with both the Israeli military’s denial of food, water, and electricity to the population of the Gaza Strip and the litany of official statements to this effect as noted above [21, 46]. Other considerations for direct attacks include Israel’s assertions that some of these civilian objects, such as hospitals, were used as military command centers. While civilian objects may lose their protection from attack only if they are being used outside their humanitarian function, to commit acts “harmful to the enemy” [47], parties must refrain from attacking a civilian object if it would firstly, cause disproportionate harm to the civilian population, or second, be carried out in a way that fails to discriminate between combatants and civilians. Ultimately, the possibility that all of the attacked sites justifiably lost their legal protections is unlikely given the lack of evidence to date, and because the most robust claim to this effect, that Gaza’s largest hospital was being used as a military “command-and-control center”, has not been proven; the purported evidence shared thus far falls far short of Israel’s initial, and specific, claims that led to the hospital being raided and attacked [48]. Independent and transparent investigations are required to assess what this data and other studies that have assessed damage to critical civilian infrastructure indicates about Israeli military intentions and practices, especially in light of the concern of war crimes committed in previous attacks on the Gaza Strip and the case of genocide brought before the International Court of Justice by South Africa [6–8, 49].

### Life-threatening conditions for mandatory evacuation

The Israeli evacuation orders for citizens living in the northern governorates (North Gaza and Gaza) into the governorates south of Wadi Gaza (Deir Al-Balah, Khan Younis, and Rafah) on 13 October 2023 were immediately and widely protested by health and humanitarian groups, with the World Health Organization calling them a “death sentence” for patients [50]. Aside from having to undertake the arduous journey, often made on foot with families carrying whatever they could of their personal belongings, evidence from this study suggests that the route itself was not safe. Our analysis finds direct targeting of infrastructure along the corridor, indicating that, despite its demarcation as an evacuation corridor, security was not guaranteed along this route, in line with on-the-ground reports from along the corridor [51, 52]. This raises additional questions about Israel’s failure to provide civilian protections, especially on routes where civilians are being mandated to go for their supposed safety.

The areas awaiting those who managed to evacuate to the south were also not spared from damage, which, combined with the overwhelming demand, led to limited services. Our data demonstrates that the evacuation orders made for civilians put them in life-threatening conditions, with direct and proximal damage to the critical civilian infrastructure in southern governorates where civilians were directed to flee. Evacuation orders by the Israeli military [51], under the auspices of civilian protection, instead placed significant strain on already overwhelmed critical civilian facilities. Health facilities in the southern governorates of Gaza reported critical overflows of traumatic and burn injuries [53], with limited capabilities to provide basic humanitarian services due to ongoing attacks on infrastructure and limited humanitarian aid creating a humanitarian catastrophe [54, 55].

### Limitations

Our analysis is based on satellite imagery-based assessments of likely damage, which lacks granular, building-by-building detail that would improve our understanding of the severity and types of damage to certain facilities and infrastructure. The data also does not differentiate between types of attacks, whether from the ground or from the air. The lack of ground truth data on locations and type of structural damage also prevents the ability to quantify the accuracy of satellite radar-based damage assessments. While theoretically difficult to differentiate between infrastructure damage caused by Israeli munitions and Hamas munitions after the initiation of a ground invasion by Israel, the unprecedented tonnage of munitions [10] used and a military campaign described as ‘the most destructive in recent history’ [56] makes the likelihood of causes of overwhelming infrastructure damage other than Israeli military munitions extremely low.

## Conclusions

The first phase of the Israeli military campaign in the Gaza Strip from 7 October through 22 November 2023 resulted in widespread damage to critical civilian infrastructure protected under IHL, including health, education, and water facilities. Spatial statistical analysis suggests widespread damage to critical civilian infrastructure that should have been provided protection under IHL. These findings raise serious allegations of Israeli military violations of IHL, especially in light of Israeli officials’ statements explicitly inciting violence and displacement and multiple widely reported acts of collective punishment against the Palestinian population in the Gaza Strip.

### Electronic supplementary material

Below is the link to the electronic supplementary material.

Supplementary Material 1

## Data Availability

Datasets used for this study may be found in Table 1
