# Supplementary material for: ‘Nowhere and no one is safe’: spatial analysis of damage to critical civilian infrastructure in the Gaza Strip during the first phase of the Israeli military campaign, 7 October to 22 November 2023
Source: Confl Health. 2024 Apr 2;18:24. doi: 10.1186/s13031-024-00580-x (PMC10985964; doi:10.1186/s13031-024-00580-x)
Supplement: Supplementary file 1 — Supplementary Material 1 [file 13031_2024_580_MOESM1_ESM.docx]

|  |  | **North Gaza** | | | | **Gaza** | | | | **Deir Al-Balah** | | | | **Khan Younis** | | | | **Rafah** | | | |
| --- | --- | --- | --- | --- | --- | --- | --- | --- | --- | --- | --- | --- | --- | --- | --- | --- | --- | --- | --- | --- | --- |
|  |  | **Total Facilities** | **Damaged** | | | **Total Facilities** | **Damaged** | | | **Total Facilities** | **Damaged** | | | **Total Facilities** | **Damaged** | | | **Total Facilities** | **Damaged** | | |
| **Buffer level (meters)** | **Facility Type** | **N** | **Any damage** | **<49%** | **>=50%** | **N** | **Any damage** | **<49%** | **>=50%** | **N** | **Any damage** | **<49%** | **>=50%** | **N** | **Any damage** | **<49%** | **>=50%** | **N** | **Any damage** | **<49%** | **>=50%** |
|  | Healthcare facilities | 17 |  |  |  | 32 |  |  |  | 18 |  |  |  | 18 |  |  |  | 12 |  |  |  |
| 0 |  |  | 15 (88.2%) | 3 (17.6%) | 12 (70.6%) |  | 24 (75%) | 8 (25%) | 16 (50%) |  | 10 (55.6%) | 6 (33.3%) | 4 (22.2%) |  | 7 (38.9%) | 5 (27.8%) | 2 (11.1%) |  | 3 (25%) | 3 (25%) | 0 (0%) |
| 25 |  |  | 16 (94.1%) | 4 (23.5%) | 12 (70.6%) |  | 27 (84.4%) | 16 (50%) | 11 (34.4%) |  | 12 (66.7%) | 9 (50%) | 3 (16.7%) |  | 9 (50%) | 8 (44.4%) | 1 (5.6%) |  | 4 (33.3%) | 4 (33.3%) | 0 (0%) |
| 50 |  |  | 16 (94.1%) | 5 (29.4%) | 11 (64.7%) |  | 29 (90.6%) | 17 (53.1%) | 12 (37.5%) |  | 14 (77.8%) | 11 (61.1%) | 3 (16.7%) |  | 12 (66.7%) | 11 (61.1%) | 1 (5.6%) |  | 5 (41.7%) | 5 (41.7%) | 0 (0%) |
|  | Education facilities | 87 |  |  |  | 199 |  |  |  | 39 |  |  |  | 85 |  |  |  | 65 |  |  |  |
| 0 |  |  | 69 (79.3%) | 17 (19.5%) | 52 (59.8%) |  | 166 (83.4%) | 51 (25.6%) | 115 (57.8%) |  | 25 (64.1%) | 18 (46.2%) | 7 (17.9%) |  | 39 (45.9%) | 28 (32.9%) | 11 (12.9%) |  | 25 (38.5%) | 19 (29.2%) | 6 (9.2%) |
| 25 |  |  | 73 (83.9%) | 29 (33.3%) | 44 (50.6%) |  | 179 (89.9%) | 79 (39.7%) | 100 (50.3%) |  | 27 (69.2%) | 22 (56.4%) | 5 (12.8%) |  | 49 (57.6%) | 43 (50.6%) | 6 (7.1%) |  | 32 (49.2%) | 26 (40%) | 6 (9.2%) |
| 50 |  |  | 81 (93.1%) | 41 (47.1%) | 40 (46%) |  | 188 (94.5%) | 90 (45.2%) | 98 (49.2%) |  | 30 (76.9%) | 28 (71.8%) | 2 (5.1%) |  | 55 (64.7%) | 51 (60%) | 4 (4.7%) |  | 38 (58.5%) | 33 (50.8%) | 5 (7.7%) |
|  | Water facilities | 33 |  |  |  | 45 |  |  |  | 21 |  |  |  | 36 |  |  |  | 17 |  |  |  |
| 5 |  |  | 23 (69.7%) | 5 (15.2%) | 18 (54.5%) |  | 29 (64.4%) | 2 (4.4%) | 27 (60%) |  | 3 (14.3%) | 0 (0%) | 3 (14.3%) |  | 6 (16.7%) | 1 (2.8%) | 5 (13.9%) |  | 3 (17.6%) | 0 (0%) | 3 (17.6%) |
| 25 |  |  | 24 (72.7%) | 1 (3%) | 23 (69.7%) |  | 36 (80%) | 8 (17.8%) | 28 (62.2%) |  | 6 (28.6%) | 3 (14.3%) | 3 (14.3%) |  | 8 (22.2%) | 3 (8.3%) | 5 (13.9%) |  | 4 (23.5%) | 2 (11.8%) | 2 (11.8%) |
| 50 |  |  | 25 (75.8%) | 4 (12.1%) | 21 (63.6%) |  | 40 (88.9%) | 14 (31.1%) | 26 (57.8%) |  | 11 (52.4%) | 8 (38.1%) | 3 (14.3%) |  | 9 (25%) | 3 (8.3%) | 6 (16.7%) |  | 4 (23.5%) | 2 (11.8%) | 2 (11.8%) |

Supplemental Table 1

Number of damaged facilities and amount of damage at each buffer level by facility type and Gaza Strip governorate
